# Hepatitis C and Hepatitis B Virus Infection: Epidemiology and Risk Factors in a Large Cohort of Pregnant Women in Lorestan, West of Iran

**DOI:** 10.5812/kowsar.1735143X.749

**Published:** 2011-09-01

**Authors:** Seyed Reza Mohebbi, Azar Sanati, Kourosh Cheraghipour, Mohammad Rostami Nejad, Hamid Mohaghegh Shalmani, Mohammad Reza Zali

**Affiliations:** 1 Research Institute of Gastroenterology and Liver Diseases, Shahid Beheshti University of Medical Sciences, Tehran, IR Iran; 2 Department of Parasitology, Lorestan Veterinary Office, Khorram Abad, IR Iran

**Keywords:** Hepatitis B, Hepatitis C, Pregnant women, Anti-HBc antibody, HBs-Ag, Anti-HCV antibody

## Abstract

**Background:**

There are little data on the prevalence of serological markers of hepatitis B and hepatitis C viruses in pregnant women in Iran.

**Objectives:**

This study was designed to determine the prevalence of hepatitis B virus (HBV) and hepatitis C virus (HCV) infection among pregnant women in Lorestan, west of Iran.

**Patients and Methods:**

Serum samples of 827 pregnant women who lived in rural (36.8%) and urban areas (63.2%) of Lorestan were collected during 2007-2008. Data were obtained through questionnaires. Samples were first screened for anti-HCV and anti-HBc by ELISA. Those who were positive for anti-HBc were tested for HBsAg.

**Results:**

Anti-HBc was found in 28 of 827 pregnant women (overall prevalence, 3.4%; 14 of 523 in urban areas, 2.7%; 14 of 304 in rural areas, 4.6%). Of the 28 positive samples, 6(0.7%) were positive for HBs-Ag. Only 2 samples (0.2%) were anti-HCV-positive.

**Conclusions:**

These results underscore the need for prenatal screening for HBV infection in pregnant women and treatment of newborns from HBsAg-positive mothers.More studies are needed to identify risk factors of HCV infection and highlight the importance of HCV screening and treatment programs.

## 1. Background 

Hepatitis B virus infection (HBV) and hepatitis C virus infection (HCV) are serious public health problems worldwide; an estimated 170 and 400 million persons suffer from these infections [1][2]. HBV and HCV are a contagious diseases in Iran that can be transmitted vertically from mothers to their neonates or horizontally by blood products and body secretions [3][4]. According to the latest data in Iran, 1.2% to 9.7% of the general population has acquired hepatitis B surface antigen (HBs Ag) (about 1.5-2.5 million people), and 0.12% to 0.89% has anti-hepatitis C virus, which corresponds to about 0.5 million chronic carriers of hepatitis C [3][5][6][7]. Chronic HBV infection at birth occurs in approximately 90% of infants who are born to HBsAg- and HBeAg-positive mothers [8]. Even if they are not infected during pregnancy, children of HBV-infected mothers have a high risk of acquiring HBV infection by horizontal transmission during the first years of life [9]. On the other hand, the range of transmission of HCV from mother to child during pregnancy is between 0% and 15% [10]. The prevalence of HBV among pregnant women worldwide is approximately 5% [11], ranging from 0.6% in low-endemic regions to > 20% in high-endemic areas in the Far East and Africa [11]. In contrast, the prevalence of HCV among pregnant women worldwide is between 1% and 8% [12].

## 2. Objectives

There are little data on the prevalence of serological markers of HBV and HCV among pregnant women in Iran. Thus, the aim of the present study was to determine the prevalence of HBV and HCV infection among pregnant women who attended the reproduction section of rural and urban health care centers in Lorestan province, west of Iran, to identify target groups for postpartum immunization.

## 3. Patients and Methods

### 3.1. Population

In this cross-sectional study, we recruited all pregnant women who attended the reproduction section of rural and urban health care centers in Lorestan province, west of Iran, between 2007 and 2008. Those who did not wish to participate in this project were excluded from study. Of 827 pregnant women, 304 (36.8%) and 523 (63.2%) samples were collected from rural and urban areas, respectively. The subjects had a history of spontaneous deliveries, miscarriages, and previous successful pregnancies. The purpose and procedures of the study were explained to all participants by a trained researcher, and written informed consent was obtained from all subjects. Participant information, including sociodemographic information, clinical manifestation, behavioral data, age, job, prison history, tattooing, and IV drug abuse, were gathered. The study was approved by the Institutional Ethics Committee of the Research Center for Gastroenterology and Liver Disease (RCGLD), Shahid Beheshti University of Medical Sciences.

### 3.2. Blood Sample

To obtain serum, blood samples were taken and centrifuged to separate the serum. Serum samples were transferred to RCGLD under cold conditions by airplane and stored at -20°C. Samples were first screened for anti-HBc and anti-HCV using commercially available ELISA kits (DIA PRO Diagnostic Bioprobes, Srl., Italy). Those who were positive for anti-HBc were tested for HBsAg (ELISA kits DIA PRO Diagnostic Bioprobes, Srl., Italy).

### 3.3. Statistical Analysis

SPSS, version 15.0, was used for descriptive statistics. We also used corrected χ² test or Fisher’s exact test to compare percentages, and P < 0.05 was considered statistically significant.

## 4. Results

Eight hundred twenty seven pregnant women with mean age of 26.1 ± 5.4 (mean ± SD) years (range: 13-42 years) and mean gestational period of 5.5 ± 2.3 months, were studied. Anti-HBc was found in 28 of the 827 women. There was no difference between mean age of anti-HBc-positive patients and -negative subjects (Table).

Of 28 anti-HBc-positive patients, 6 (0.7%) were positive for HBs-Ag; this difference was not significant between urban and rural populations. Of 827 pregnant women, 2 samples (0.2%) were anti HCV-positive. These patients were also HBs-Ag-positive and from urban areas, and none had a history of miscarriage (Table). No anti-HBc-, HBs-Ag-, or anti-HCV-positive subject had a history of tattooing or being in prison or was an IV drug user. There were no significant differences in age, history of miscarriage, occupation, education level, income, or other general socioeconomic parameters between HBs-Ag positive and negative as well as anti-HCV positive and negative (P > 0.05).

**Table:** Demographic Feature of Study Population

	**No, (n = 827)**	**Anti-HBc positive, No. (%), (n = 28)**	***P* value**	**HBs Ag positive, No. (%), (n = 6)**	***P* value**	**Anti-HCV positive, No. (%), (n = 2)**	***P* value**
Age,y			0.81		0.89		0.56
≤ 20	127	4 (3.1)		1(0.8%)		1 (0.8%)	
21-30	526	20 (3.8)		3(0.6%)		1(0.2%)	
≥31	174	4 (2.3)		2(1.1%)		0 (0%)	
Location			0.14		0.86		0.53
Urban	523	14 (2.7)		4(0.8%)		2 (0.4%)	
Rural	304	14 (4.6)		2(0.7%)		0 (0%)	
Educational level			0.87		0.57		0.63
Illiterate	41	1(2.4)		1(2.4%)		0 (0%)	
Under Diploma	308	8(2.6)		3 (1%)		0 (0%)	
Diploma	357	14(3.9)		1(0.3%)		2 (0.6%)	
University Degree	121	5(4.1)		1(0.8%)		0 (0%)	
Occupation			0.62		0.63		0.78
Housekeeper	796	28(3.5)		6(0.8%)		2 (0.3%)	
Employee	31	0(0)		0 (0%)		0 (0%)	
History of miscarriage			0.69		0.49		0.83
Yes	130	6(4.6)		2(1.5%)		0 (0)	
No	697	22(3.2)		4(0.6%)		2 (0.3%)	

## 5. Discussion 

Chronic liver diseases are an important health challenge worldwide, wherein HBV or HCV infection is the main cause of liver insufficiency with different verities in all over the world [13]. The seroprevalence of HBsAg in our study (0.7%) was lower than the rate of HBsAg positivity in pregnant women in different parts of Iran, including Zahedan (6.5%) [14], Qazvin (3.4%) [15], Bonab (3.2%) [16], Kerman (2.3%) [17], Ahwaz (1.7%) [18], Rafsanjan (1.3%) [19], and Yazd (0.84%) [20], but was higher than in Kashan (0.35%) [21]. Our rates are also lower than those for North Africa and Middle Eastern countries, in which the prevalence of HBsAg among pregnant women is 1.4% to 20% [14]. HBsAg positivity among pregnant women in urban areas in the US is 0.1% in Hispanics to 1% in non-Hispanic blacks; notably, HBsAg positivity is more prevalent among Asians (5.8%) [22].

Our study shows that the seroprevalence of anti-HCV is 0.2%, which is lower than a survey by Motlagh ME et al. in Ahvaz (6.25%) [23], while our study result was compatible with those of Brazilian (0.2%) and Canadian studies (0.5%) [24][25]. The limitations of our study are that we did not investigate a similar population of nonpregnant women to compare the findings and surveyed a single city in this province.

In conclusion, HBV and HCV are not prevalent among pregnant women in Lorestan province compared with the rest of the country. However, this low prevalence justifies the establishment of a national program for regular screening of all pregnant women. Because most pregnant women are asymptomatic and clinically disease-free, they might not be aware that they are infected and can transmit their infection to newborns.
